# The prevalence, severity, and risk factors for dry eye disease in Dubai – a cross sectional study

**DOI:** 10.1186/s12886-021-01978-4

**Published:** 2021-05-17

**Authors:** Sarah Alkabbani, Lakshmanan Jeyaseelan, Anupama P. Rao, Sandeep P. Thakur, Pramod T. Warhekar

**Affiliations:** 1 College of Medicine, Mohammed Bin Rashid University of Medicine and Health Sciences, Dubai, UAE; 2 Department Of Ophthalmology, Mediclinic City Hospital, Dubai Healthcare City, P O Box 251103, Dubai, UAE

**Keywords:** Dubai, Prevalence, Dry eyes, Associated factors, Severity, OSDI, Questionnaire, Cross-sectional, Dry eye disease, United Arab Emirates

## Abstract

**Background:**

The prevalence of dry eye disease is increasing globally and requires the attention of healthcare professionals as it worsens patients’ quality of life. No published studies on the epidemiology of dry eyes have been found in Dubai.

**Purpose:**

To describe the epidemiology, prevalence, severity, and associated factors of dry eyes in Dubai, United Arab Emirates, in 2019.

**Methods:**

This was an analytical, cross-sectional, survey-based study. An online survey was distributed by email to Mohammed Bin Rashid University students, staff, and faculty and to the staff at Mediclinic City and Parkview Hospitals in Dubai, United Arab Emirates, from April–June 2019. The survey included demographic questions and the Ocular Surface Disease Index (OSDI).

**Results:**

The survey was completed by 452 participants; the majority were females (288/452; 63.7 %). The prevalence of dry eyes in Dubai was estimated to be 62.6 % (283/452), with severely dry eyes being the most prevalent (119/283; 42 %). Females, high daily screen time (> 6 h), and the use of contact lenses were found to be associated with dry eyes (*P*-value < 0.05, 95 % confidence interval). Age was found to be negatively correlated with prevalence of dry eyes. Exposure to smoking/shisha, history of eye injury/surgery, and nationality were not associated with dry eyes.

**Conclusions:**

This is the first cross-sectional study to investigate the prevalence of dry eyes in Dubai (62.6 %). The majority of participants had severe dry eyes symptoms. Severely dry eyes were more common among females and users of contact lenses.

**Supplementary Information:**

The online version contains supplementary material available at 10.1186/s12886-021-01978-4.

## Key messages

**What is already known about this subject?**

- Dry eye disease is a growing health problem that worsens a patient’s quality of life across physical, psychological, and social dimensions [1]. Validated questionnaires are used to estimate the severity of dry eyes [2].
- Dry eyes can be a manifestation of underlying illnesses, such as autoimmune disease [3].

**What does this study add?**

- This is the first analytical, cross-sectional study to be conducted in Dubai to estimate the prevalence of dry eyes as 62.6%. It classifies dry eyes into categories by severity and tests for any associations with possible contributing factors.

**How might this study have an impact on public health and clinical practice?**

- The study raises awareness about the burden of dry eyes in Dubai. It informs healthcare professionals that a prompt diagnosis of dry eyes is important for patients who visit ophthalmology clinics with these symptoms.
- The study highlights the need to educate the public about the importance of screening for dry eyes and the need for future research about factors associated with this condition.

- This is the first analytical, cross-sectional study to be conducted in Dubai to estimate the prevalence of dry eyes as 62.6 %. It classifies dry eyes into categories by severity and tests for any associations with possible contributing factors.

## Introduction

According to the International Dry Eye Workshop, dry eye disease is a multifactorial disease of the tears and ocular surface that results in symptoms of discomfort, visual disturbance, with potential damage to the ocular surface [4]. The prevalence of dry eye disease is increasing globally and requires the attention of ophthalmologists because it worsens patients’ quality of life in different dimensions [1]. The condition is highly prevalent and affects approximately one in five adults [3]. The tear film is an important part of the ocular surface that provides lubrication to the eyes, supplies nutrition and oxygen, and supports the elimination of debris from the eye’s surface [5]. In addition, early detection of dry eyes is important because it can indicate the presence of systemic diseases, such as systemic lupus erythematosus, rheumatoid arthritis, and Sjogren’s syndrome [3]. Some studies have found an association between smoking, wearing contact lenses, the amount of daily screen time, and dry eyes [6, 7]. Although dry eyes are common, the condition is often underdiagnosed, which may be due to variations in presentation and symptoms [8]. Similarly, sometimes it is difficult to treat dry eye disease due to its multifactorial etiologies [8].

In China, the prevalence of dry eyes was 17 % [9]. Similarly, Spain had a low incidence of dry eyes (11 %) [10], compared to northern India (32 %) [6]. There are few published articles related to the prevalence of dry eyes in the United Arab Emirates (UAE), and none were found in Dubai. Therefore, this study is the first to estimate both the scope and the burden of the disease in Dubai. It is vital to identify the prevalence and severity of dry eyes to suggest future public health measures that can raise awareness and explore ways to alleviate dry eyes.

The objective of the study is to estimate the prevalence of dry eyes, the severity, and find the associated risk factors for dry eyes in Dubai. The following possible factors were investigated, and their prevalence was included: age, sex, use of contact lenses, daily amount of screen time, exposure to smoking/shisha, humidity, history of eye surgery/injury, and nationality. Shisha is a famous smoking apparatus in the Middle East, which includes burning wood or coal to heat up flavored tobacco. The smoke is inhaled through a specific mouthpiece fitted into a pipe.

## Subjects and methods

The study was conducted and reported according to the STROBE guidelines [11]. This is an analytical, cross-sectional study that was carried out at Mohammed Bin Rashid University(MBRU), Mediclinic City Hospital, and Mediclinic Parkview Hospital, in Dubai, UAE. Participants included first-year, second year, and third-year medical students, dental students, staff, and faculty at MBRU, and the staff of Mediclinic City Hospital and Mediclinic Parkview Hospital. The inclusion criteria were as follows:16 years of age or older, residents of the UAE, Faculty, staff, and students at MBRU, Staff at Mediclinic City Hospital or Mediclinic Parkview Hospital. The exclusion criteria included unwillingness to provide informed consent, English illiteracy, and inability to access the internet.

Quantitative variables included age (years) and the Ocular Surface Disease Index (OSDI) score. This score ranges from 0 to 100, with 0being normal and 100 indicating severely dry eyes. Scores for each of the 12 OSDI questions range from 0 to 4, where 0 corresponds to “none of the time” and 4 corresponds to “all of the time” (Additional file 1). If someone’s OSDI score was > 12 then the subject was defined to have dry eyes. The OSDI score is calculated using the following formula [2]:

$$OSDI=\frac{\left(sum of scores\right) x 25}{\left(number of questions answered\right)}$$

Categorical nominal variables included sex, nationality, use of contact lenses, and any prior history of eye surgery or injury. Categorical ordinal variables included the average daily screen time (hours), which was divided into groups as follows: (1) less than three hours, (2) three to six hours, (3) More than six hours. The severity of dry eyes (mild, moderate, or severe) was classified according to the OSDI score. A score between 13 and 22 is considered as mild, 23–32 is moderate, and 33–100 is seen as severe.

Primary data were collected using an online survey that was distributed by email to the sample population. The questionnaire contains the OSDI questionnaire, which is a standardized questionnaire for the assessment of the severity of dry eyes [2]. The OSDI questionnaire is a reliable method that can be used to differentiate between healthy individuals and those with dry eyes [5]. Some literature reviews have found that sex (female) and certain medications may cause dry eyes [12]. Therefore, additional questions were added about demographics and other potentially associated factors, such as sex, age, nationality, use of contact lenses, exposure to smoking/shisha, exposure to humidity by working outdoors, average daily screen time, a history of eye surgery/injury (both physical and chemical), and any current use of medication where applicable [6].

Potential types of bias included recall bias, response bias, temporal bias, sampling bias, and questionnaire bias. The incidence of questionnaire bias was reduced by using the OSDI standardized questionnaire [2]. The OSDI is validated and considered to have a good to excellent reliability, which helps to reduce survey bias [2]. Simple terminology was also used to reduce any potential response bias.

Convenience sampling was carried out to select the study sample. Bukhari et al. [13] reported that the prevalence of Dry Eyes was 93.2 % based on 251 subjects in Saudi Arabia^. ^Assuming that Dubai will also have similar prevalence, and to estimate this with the precision of 2.5 % with 95 % Confidence Interval and with 10 % drop out, we need to study about 450 subjects.

The continuous variables such as age (years) and screening time were categorised. That is, age was categorised as < 19, 20–29, 30–39 and 40 plus years. Similarly, the screen time of cell phones and/or laptop was categorised as < 3, 3–6 and > 6 h. The outcome was categorised as normal if the OSDI score was less than or equal to 12 and having got dry eyes if the score was greater than 12. Logistic regression analysis was done with log link function as the prevalence of Dry Eye was > 10 %. Therefore, the measure of effect is Risk Ratio. Data was entered in SPSS 24.0 and analysed using STATA software 16.0 [14]. The statistical significance was fixed at 5 % level. The goodness of fit of the logistic regression model was assessed using Hosmer and Lemeshow Statistic.

 Ethical approval from the MBRU Institutional Review Board has been granted (MBRU-IRB-SRP-64-2017). This study adhered to the tenets of the Declaration of Helsinki. No personal identifiers were obtained, and the data were kept confidential and treated anonymously. The survey contained forced-answer questions to ensure that there were no missing data to complicate the calculation of the OSDI score and the following analysis. A participant information sheet was attached to the beginning of the survey that emphasized voluntary participation (Additional file 1).

## Results

Table 1 presents the distribution of demographic characteristics and the baseline risk variables for the 452 subjects studied along with the prevalence of Dry Eye (DE) with 95 % CI. The overall prevalence of Dry Eye was 62.6 % (95 % CI: 58.1,67.0). The median (IQR) for OSDI was 18 (8,33). Of the 452 subjects studied, 164 (36.3 %) and 288 (63.7 %) were males and females, respectively. The prevalence of DE was 51.8 % (44.0, 59.0) and 68.8 % (63.3, 74.1 %) for males and females respectively. Thus, the females had higher prevalence as compared to males.

**Table 1:** The Baseline Distribution and Prevalence with 95% CI for Dry Eyes

	Dry Eye						
	Total		Yes		No		Prevalence (%) (95% CI)
	No	%	No	%	No	%	
**Overall**	452	100%	283	62.6	169	37.4	62.6 (58.1, 67.0)
**Sex**							
Female	288	63.7%	198	68.8%	90	31.3%	68.8 (63.3, 74.1)
Male	164	36.3%	85	51.8%	79	48.2%	51.8 (44.0, 59.0)
**Age (years)**							
17-19	91	20.2%	59	64.8%	32	35.2%	64.8 (55.0, 74.6)
20 - 29	85	18.8%	55	64.7%	30	35.3%	64.7 (54.5, 74.8)
30-39	157	34.8%	97	61.8%	60	38.2%	61.8 (54.1, 69.3)
>=40	118	26.2%	71	60.2%	47	39.8%	60.2 (51.3, 69.0)
**Nationality**							
UAE	73	16.2%	43	58.9%	30	41.1%	58.9 (47.6, 70.1)
Others	379	83.8%	240	63.3%	139	36.7%	63.3 (58.4, 68.1)
**Contact Lens**							
Yes	80	17.7%	63	78.8%	17	21.3%	78.8 (69.7, 87.7)
No	372	82.3%	220	59.1%	152	40.9%	59.1 (54.1, 64.1)
**Eye Surgery**							
Yes	41	9.1%	26	63.4%	15	36.6%	63.4 (48.6, 78.1)
No	411	90.9%	257	62.5%	154	37.5%	62.5 (57.8, 68.8)
**Screen Time**:							
< 3 h	44	9.7%	22	50.0%	22	50.0%	50.0 (35.2, 64.7)
3-6 h	143	31.6%	87	60.8%	56	39.2%	60.8 (52.2, 68.8)
> 6 h	265	58.6%	174	65.7%	91	34.3%	65.6 (59.9, 71.3)
**Exposed to :**							
None	372	82.3%	228	61.3%	144	38.7%	61.2 (56.3, 66.2)
Smoking/ Shisha	71	15.7%	48	67.6%	23	32.4%	67.6 (56.7, 78.4)
Working outdoors	9	2.0%	7	77.8%	2	22.2%	77.7 (50.6, 100)

One fifth of the subjects studied were aged 17–19 years, and about the same were from 20 to 29 years as well. About 35 and 25 % were from 30 to 39 and greater than 39 years old, respectively. The prevalence in the teenagers was 64.8 % (55, 74.6 %). The prevalence of DE for the subjects aged 20–29 and 30–39 years was 65 % (54.5, 74.8) and 61.8 % (54.1, 69.3) respectively. However, the subjects aged greater than 40 years had the lowest prevalence 60.2 % (51.3, 69.0) as compared to others. That is, there was a negative correlation between age and prevalence of DE. About 60 % of the subjects studied were from UAE while the rest are from other countries. The prevalence of DE in UAE participants was 58.9 % (47.6, 70.1), while it was 63.3 % (58.4, 68.1) in other nationalities.

About 18 % have used contact lenses in the study. Of them, the prevalence of DE was 78.8 % (69.7, 87.7), while this was 59.1 % (54.1, 64.1) in the contact lenses nonusers. The prevalence of eye surgery in the study was 9.1 %. Of them the prevalence of DE was 63.4 % (48.6, 78.1). However, about 10 % of the subjects have screen time less than 3 h, while 60 % of the subjects have screen time more than 6 h. About one third had an average of for 3–6 h of screen time. As the screen time increased, the prevalence of DE also increased. The prevalence was 50 % percent for the subjects who have screen time for less than 3 h while this was 66 % in the group who have screen time for more than 6 h. About 16 % of the subjects have smoked or used Shisha and only 2 % worked outdoors. The prevalence DE was 67.6 % (56.7, 78.4) in the smoking or using Shisha group.

Table 2 presents the distribution of severity of DE by age, sex, and nationality. The females had about 31 % severe form of DE, while this was about half among males, that is (17.7 %). The compliment of this trend was observed in the normal group of DE. That is more males were normal. The difference by sex was statistically significant (*p* < .001). We found a negative correlation between age and the prevalence of DE. As for nationality and history of eye surgery, no significant association was found between them and prevalence of DE.

**Table 2:** The distribution of Severity by Age, Sex and Nationality

	Severity of Dry Eye								*P* Value
	Normal		Mild		Moderate		Severe		
	No	%	No	%	No	%	No	%	
**Sex:**									
Female	90	31.3%	62	21.5%	46	16.0%	90	31.3%	<.001
Male	79	48.2%	33	20.1%	23	14.0%	29	17.7%	
**Age (Years)**									
17-19	32	35.2%	20	22.0%	15	16.5%	24	26.4%	.809
20 - 29	30	35.3%	15	17.6%	19	22.4%	21	24.7%	
30-39	60	38.2%	33	21.0%	21	13.4%	43	27.4%	
40 +	47	39.8%	26	22.0%	14	11.9%	31	26.3%	
**Nationality**									
UAE	30	41.1%	12	16.4%	10	13.7%	21	28.8%	.676
Others	139	36.7%	83	21.9%	59	15.6%	98	25.9%	

Table 3 presents the risk ratio, 95 % CI and the p value of logistic regression analysis. The Females had about 2 (1.3, 3.1) times significantly higher risk as compared to males after adjusting for other risk factors (*p* = .001). Similarly, the subjects who have used contact lens had 2.5 (1.4, 4.6) times significantly higher risk as compared to who did not use contact lens (*p* = .003). The subjects who had screen time for more than 6 h had 2.3 (1.1, 4.5) times significantly higher risk for DE as compared to subjects who have screen time for less than 3 h (*p* = .017).

**Table 3:** Results of Logistic Regression analysis

	Risk Ratio	95% C.I.		***P*** value
		Lower	Upper	
**Age (years):**				
17-19	1.0			
20-29	1.221	.633	2.353	.552
30-30	1.119	.634	1.976	.699
>= 40	1.434	.763	2.696	.263
**Female**	2.062	1.350	3.150	.001
**Contact Lens User (yes)**	2.51	1.4	4.6	.003
**Eye Surgery (Yes)**	1.073	.534	2.157	.844
**Screening Time (hours):**				
<3 h	1.0			
3-6	1.697	.826	3.486	.150
> 6 h	2.300	1.161	4.557	.017
**Exposed to :**				
None	1.0			.389
Smoking / Shisha	1.371	.778	2.416	.275
Work Outdoors	2.124	.404	11.183	.374

## Discussion

This is the first study to report the epidemiology of dry eyes in Dubai. The prevalence of dry eyes from April–June 2019 was 62.6 % in our 452 participants. Females and participants who wore contact lenses reported more severely dry eyes than any other group. High daily screen time (more than 6 h) was found to be associated with a higher mean OSDI score. A negative correlation was found between age and prevalence of dry eyes, that is as age increases the prevalence of dry eyes decreases. No significant difference was found in the mean OSDI score between Emiratis and expatriates.

We have used logistic regression analyses to adjust for the confounder such as age and sex of the subjects. We have used log link instead of logit link as the prevalence of Dry Eyes was over 10 %. Therefore, the effect measure that we get is risk ratio and not odds ratio. Also because we do have enough power to do this analyses. As a result of multivariable analyses, the risk factors are very robust and reliable.

The prevalence of dry eyes reported by our study (62.6 %)was similar to that found in the population of the Eastern Province of Saudi Arabia(65.4 %) [15] and to the Jordanian population (59 %) [16]. However, Alshamrani et al.reported an incidence of 32.1 % in Saudi Arabia, which was significantly lower than the prevalence reported by our study [17]. A higher prevalence of 93.2 % was also calculated in the population of Jeddah, Saudi Arabia [13]. This discrepancy might be due to the different techniques used to diagnose dry eyes.

It is interesting to note that the Eastern Province of Saudi Arabia had a similar prevalence of dry eyes as that found in Dubai and is also near the UAE, while Jeddah is in the Western Province and had a significantly higher prevalence of dry eyes. The prevalence may also have varied partly due to the differing definition of dry eyes between studies [5].

In China, the prevalence of dry eyes was 17 %, which is significantly lower than the rate found in our study [9]. Similarly, Spain had a lower incidence of dry eyes (11 %) compared to our Dubai study population [10]. This variation may be influenced by factors that could include climate and lifestyle differences between the locations [8].

Our study found similar risk factors such as sex (female), use of contact lenses, daily screen time, and dry eyes as to Titiyal et al. [6]. A high amount of screen time is associated with dry eyes due to the decrease in the blink rate in screen users, which disturbs the tear film [18]. Higher estrogen concentrations in females may affect the production of the tear film [19, 20]. Furthermore, since dry eye disease is associated with autoimmune diseases, it is more likely to occur in females [19, 20]. The present study also found an association between using contact lenses and the OSDI score. Contact lenses decrease the goblet cell density and result in conjunctival metaplasia, which can disrupt the tear film [7].

Smoking predisposes people to dry eyes by decreasing the tear film break up time [21]. However, no association between smoking and dry eyes was found in this study. Only16 % (71/452) of our participants smoked or were exposed to smoking, while Al-Houqani et al. found a higher prevalence of smoking in the UAE (42 % of males, 6 % of females) [22]. This discrepancy can be explained by the study sample, which is limited to healthcare professionals who are less likely to smoke because of their health education. Our participants spent most of their time indoors and therefore had a low exposure to smoking, which might explain the absence of an association between smoking and dry eyes in the current study. Participants were also exposed to air-conditioners, which is a confounding factor because low humidity is a risk factor for dry eyes [18].

We found that older participants have lower prevalence of DE. This could be because the elderly tend to have less daily screen time due to eyesight abnormalities or ocular diseases. Furthermore, not all people age in the same way, and the influence of age on dry eyes is multifactorial [23].

About 18 % of the participants used medication or supplements, with the most common being eye drops for dryness, which indicated that the sample participants were aware of the dry eye condition. Some participants were assumed diabetic as they were currently taking diabetic medication. Antihistamines, cholesterol-lowering agents, and oral contraceptives were also reported by few participants and are known to cause dry eyes [5].

The high prevalence of dry eyes indicates the need for early diagnosis and treatment before complications occur [5]. This widespread prevalence emphasizes the role of the general practitioner to refer patients to ophthalmologists, especially if they have autoimmune diseases or are taking long-term medications. Public health officials should aim to educate the population that dry eyes may be an indicator of other chronic diseases [13]. Future studies should recruit a larger sample that is more representative of the population of Dubai. In addition, it would be good idea to combine the clinical diagnosis of dry eyes with a validated questionnaire in future studies.

The respondents come from 47 nationalities, which reflects the cultural diversity in Dubai. The ages ranged from 17 to 67 years, which helps to improve the generalizability of the sample. The calculated sample size of 440was achieved with 452 respondents, which increases the confidence level regarding the power of the test.

The survey used the OSDI questionnaire, which is a standardized and reliable questionnaire that is used by many researchers [2]. The OSDI provides a relatively objective evaluation of the symptoms of dry eyes, and it is used in clinical treatment trials, which helps to eliminate the possibility of survey bias [8].

Selection bias is a limitation of the study as it was limited to English-literate respondents who could access the internet, which limits the generalizability of our results. Due to feasibility issues, we were not able to choose the community to do the study. Though the sampling frame is available from MBRU, Mediclinic and other hospitals, we were not able to apply the random sampling method because of subject’s availability at various timings.

Our study was also geographically limited and does not represent the entire population of Dubai. Since the sample represents a population that is highly educated and has a medical background, there is huge sampling bias which affects the generalizability of the study. We have discussed these limitations in relation to risk factors such as contact lens and screen time, which are more common in the educated group rather than the general population. Therefore, this prevalence may be an over estimation rather than under estimation.

In addition, we asked respondents about any current medications to identify any comorbidities, but this method was unreliable because some drugs are used to treat multiple conditions.

Our study relied solely on the OSDI questionnaire to predict the diagnosis of dry eyes and did not use clinical tests for confirmation. The survey also was based on respondent recall of events from the past week, which predisposes the results to recall bias.

## Conclusions

This study was the first cross-sectional study conducted in Dubai to determine the prevalence of dry eyes, which was found to be 62.6 % in Dubai in 2019. We identified an association between female sex, a high amount of average daily screen time, the use of contact lenses, and dry eyes. However, age, a history of eye injury, and exposure to smoking/shisha were not found to be associated with dry eyes. Severely dry eyes are more common in females and in people who regularly wear contact lenses. Our findings may help raise awareness about the burden and magnitude of dry eyes in places similar to Dubai, UAE. We recommend future studies to estimate the prevalence dry eyes using a more representative sample of the population of Dubai. The OSDI questionnaire can be combined with objective tests such as Schirmer’s test.

## Supplementary Information

**Additional file 1.**

## Data Availability

The datasets used and/or analysed during the current study are available from the corresponding author on reasonable request.
