# Supplementary material for: The prevalence, severity, and risk factors for dry eye disease in Dubai – a cross sectional study
Source: BMC Ophthalmol. 2021 May 17;21:219. doi: 10.1186/s12886-021-01978-4 (PMC8127306; doi:10.1186/s12886-021-01978-4)

Original Article

The Prevalence, Severity, and Risk Factors for Dry Eye Disease in Dubai – A Cross sectional study

**Authors**

Sarah Alkabbani^1^

Lakshmanan Jeyaseelan^1^

Anupama P. Rao^1,2^

Sandeep P. Thakur^2^

Pramod T. Warhekar^1,2^

^1^ Mohammed Bin Rashid University of Medicine and Health Sciences, College of Medicine, Dubai, (UAE)

^2^ Department Of Ophthalmology, Mediclinic City Hospital, Dubai Healthcare City, Dubai, (UAE)

**Correspondence to** Dr. Pramod T. Warhekar, Department of Ophthalmology, Mediclinic City Hospital, P O Box 251103, Dubai (UAE); [pramod.warhekar@mediclinic.ae](mailto:pramod.warhekar@mediclinic.ae); [warhekar@gmail.com](mailto:warhekar@gmail.com), 00971555946055

**Appendix**

**Questionnaire**

1. Age:
2. Gender:
3. Nationality:
4. Do you find it difficult to open your eyes in the morning due to dryness?

- Yes
- No

1. Do you use contact lenses?

- Yes
- No

1. Do you have a history of eye surgery or injury (including chemical injury)?

- Yes
- No

1. Current use of medication if any:
2. Are you exposed to any of the following? Please tick

- Smoking/shisha
- Working outdoors
- None of the above

10) What is your approximate screen time per day (Mobile, Laptop, and TV all together)?

- Less than 3 hours
- 3-6 hours
- More than 6 hours


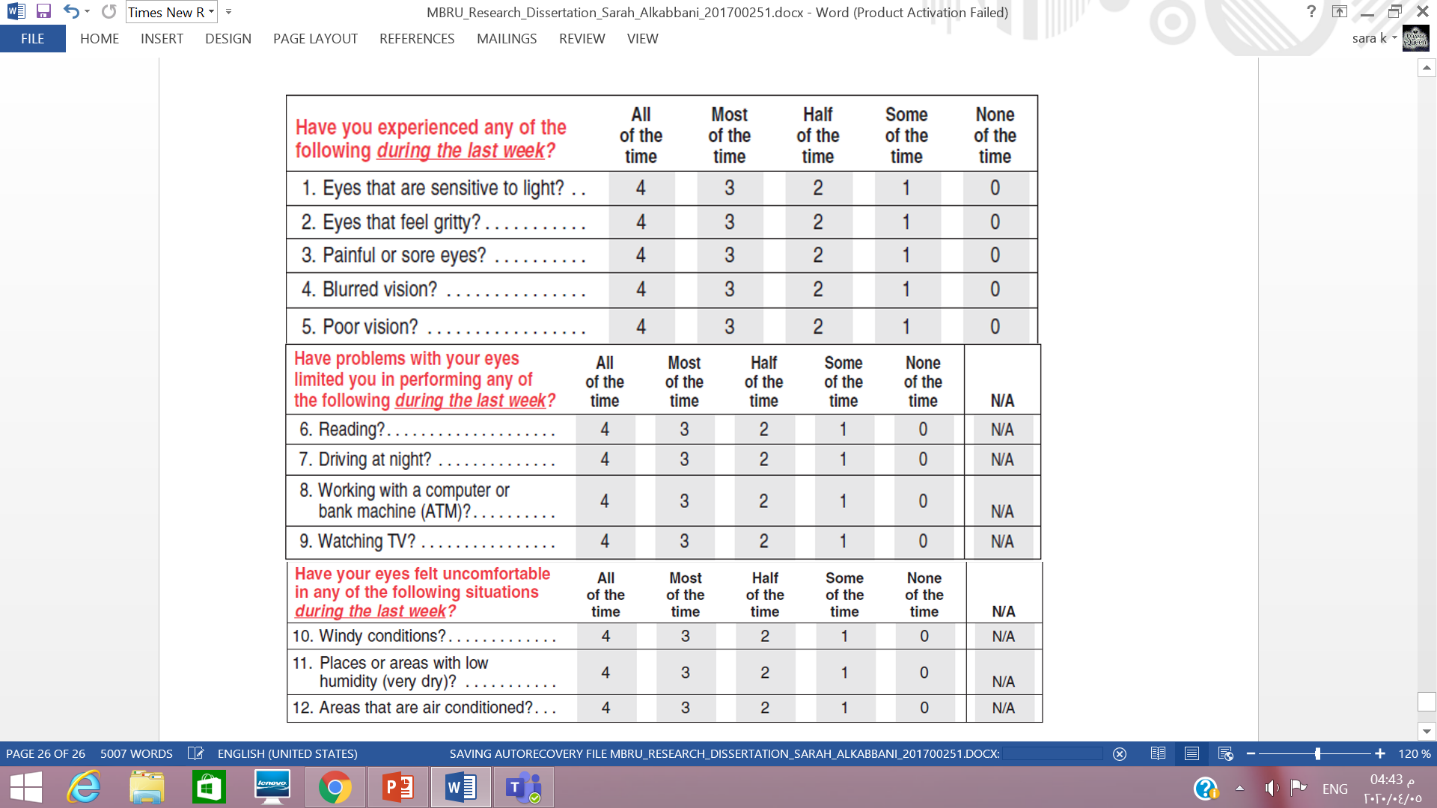

Supplement: Supplementary file 1 — Additional file 1. [file 12886_2021_1978_MOESM1_ESM.docx]
